# A QoS Aware Resource Allocation Strategy for 3D A/V Streaming in OFDMA Based Wireless Systems

**DOI:** 10.1155/2014/419236

**Published:** 2014-08-28

**Authors:** Young-uk Chung, Yong-Hoon Choi, Suwon Park, Hyukjoon Lee

**Affiliations:** College of Electronics and Information Engineering, Kwangwoon University, Seoul 139-701, Republic of Korea

## Abstract

Three-dimensional (3D) video is expected to be a “killer app” for OFDMA-based broadband wireless systems. The main limitation of 3D video streaming over a wireless system is the shortage of radio resources due to the large size of the 3D traffic. This paper presents a novel resource allocation strategy to address this problem. In the paper, the video-plus-depth 3D traffic type is considered. The proposed resource allocation strategy focuses on the relationship between 2D video and the depth map, handling them with different priorities. It is formulated as an optimization problem and is solved using a suboptimal heuristic algorithm. Numerical results show that the proposed scheme provides a better quality of service compared to conventional schemes.

## 1. Introduction

Recent advances in video technology have made three-dimensional audio/video (3D A/V) services technically feasible. 3D A/V has emerged to provide more immersive experiences compared to conventional two-dimensional (2D) video. It allows viewers to feel a compelling sense of physically real spaces. Therefore, it is expected to be a “killer app” for smart-phones and corresponding broadband wireless systems.

3D A/V is generated by multiview images, which is obtained in two ways: through multiview cameras [1, 2] and with a depth-image-based rendering (DIBR) technique [3–7]. Multiview cameras involve a number of cameras to obtain images according to users' viewpoints. However, these types of cameras require complicated coding and careful transmission techniques because the amount of data increases proportionally to the number of cameras.

Instead of using raw data of multiviewed images, the DIBR technique makes use of two types of images: 2D texture images and synchronized depth maps. This is known as the video-plus-depth concept, which was introduced by the advanced three-dimensional television system technologies (ATTEST) project [8]. These two types of image streams are separately encoded into the base layer and the enhancement layer. The DIBR technique can reconstruct and render 3D video from these two data streams. Because the required data size and the degree of processing complexity are relatively low, the DIBR technique is considered as suitable for a 3D A/V system.

As mentioned above, the 3D A/V service is expected to become widely popular on smart-phones and corresponding broadband wireless systems based on the orthogonal frequency division multiple access (OFDMA). OFDMA technology is known to be suitable for next-generation broadband wireless systems because high-speed data transmission is possible under limited radio resources. However, the size problem is still severe in OFDMA-based wireless systems because the limited radio resources represent a fundamental problem of current wireless systems. Though the video-plus-depth concept based on the DIBR technique can reduce the amount of data compared to the multiview camera method, the amount of 3D A/V traffic is still large enough such that it can hardly be transmitted over a wireless system in real-time. Therefore, the efficient resource allocation of radio resources is important to guarantee the QoS of a 3D A/V service.

In OFDMA-based wireless systems, resource allocation is performed by means of a scheduling procedure. There have been many scheduling algorithms for wireless systems. The maximum carrier-to-interference ratio (Max C/I) [9], round-robin (RR) [10], proportionally fair (PF) [11], and fast fair-throughput (FFTH) [12] scheduling algorithms are the most commonly used types. The Max C/I algorithm provides throughput maximization and the RR algorithm achieves the optimal level of fairness. The PF and the FFTH algorithms provide a good balance between system throughput and fairness, but they do not take the QoS into account. Several scheduling algorithms have been studied to guarantee QoS for end users. The modified largest weighted delay first (MLWDF) [13] and the exponential rule (EXP) [14] algorithms are the most popular types. They consider both the maximum allowable delay and the instantaneous channel rate. However, little work has been carried out to find an optimal resource allocation scheme that considers the QoS of a 3D A/V service.

In this paper, we propose a resource allocation scheme for wireless transmissions of 3D A/V traffic. We focus on a QoS guarantee for 3D A/V services in the proposed scheme. To do this, we adopt a concept which uses a base layer and an enhancement layer for 3D A/V traffic, as introduced by video-plus-depth concept. This concept processes 3D traffic more efficiently.

The rest of this paper is organized as follows: first, we give an overview of the system environment in which the proposed scheme is adopted in Section 2. In Section 3, we introduce the proposed resource allocation scheme and formulate it as an optimization problem. Then, we give a detailed explanation of a suboptimal heuristic algorithm to solve the optimization problem. In Section 4, we describe the simulation environment and evaluate the performance of the proposed scheme based on several numerical results. Finally, we conclude the paper in Section 5.

## 2. System Description

This section gives an overview of the system environment in which the proposed resource allocation scheme is adopted. It begins with an introduction of the overall system architecture of 3D video over wireless systems. Also, a detailed description of video-plus-depth 3D video is discussed.

### 2.1. 3D Video over Wireless Systems

**Figure 1:**
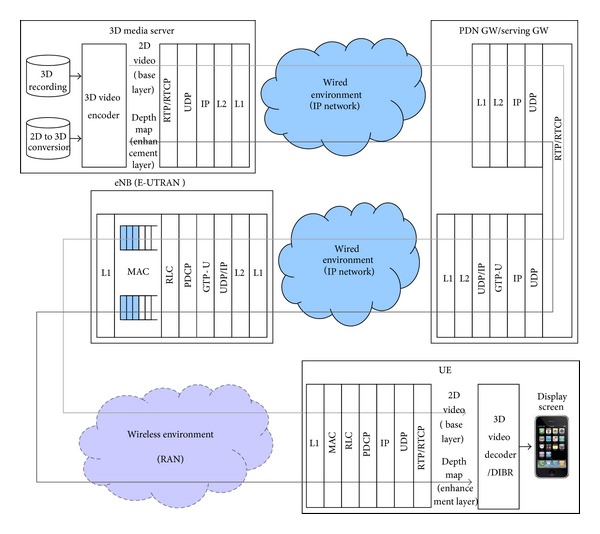
System architecture of 3D A/V over wireless systems.

Figure 1 shows the overall system architecture of 3D video over wireless systems as considered in this paper. The system consists of four parts, denoted here as a 3D media server, a packet data network gateway (PDN GW)/serving GW, an enhanced node-B (eNB)/enhanced universal terrestrial radio access network (E-UTRAN), and the user equipment (UE). The 2D video and its associated depth map are obtained from a depth camera system or with a 2D-to-3D conversion method. These data are separately encoded as the base and the enhancement layers. They are transmitted as separate streams of media data through a single connection. Before transmitting over the IP network, these media data are packetized into individual RTP packets.

The media server sends RTP packets to the wireless system through an IP network. In this paper, we consider wireless systems based on the orthogonal frequency division multiple access (OFDMA), such as the long-term evolution (LTE), LTE-advanced (LTE-A), IEEE 802.16e, IEEE 802.16 m, and other technologies. In Figure 1, we show an example of the system architecture, which adopts the radio access network (RAN) of the LTE-A systems. As shown in the figure, LTE-A system uses the PDN GW/serving GW, eNB, and UE components. This system is connected to a 3D media server through IP networks.

The PDN GW or serving GW connects the IP network and the LTE-A system. The RTP packets of the base layer and the enhancement layer data come into the LTE-A system through the general packet radio service (GPRS) tunneling protocol (GTP). GTP is an IP-based protocol which is used in the universal mobile telecommunications system (UMTS) network. GTP-U in Figure 1 is used to carry user data within the GPRS core network and between the RAN and the core network. The carried user data has usually IP or PPP format. In our system, the user data has IP format.

eNB receives user data through GTP-U/UDP/IP and then sends the data to the UE through the RAN. Each instance of data is passed through the packet data convergence protocol (PDCP) and the radio link control (RLC), after which it is segmented and encapsulated as one or more medium access control (MAC) frames and inserted into MAC buffer. The base layer traffic and the enhancement layer traffic are inserted into logically separated MAC buffers. In the eNB MAC layer, radio resources are allocated by scheduling. After resource allocation by scheduling, eNB repeatedly reads MAC frames from the MAC buffer and sends them to the UE through the RAN.

Upon receiving the MAC frames, the UE reassembles and decapsulates them as RTP packets through RLC/PDCP/IP/UDP layers. The RTP packets are combined into the base layer and the enhancement layer data. Each layer of data is decoded so as to recover the depth map and the 2D video, respectively. Finally, they are converted into a 3D video stream and displayed by the display screen of the UE.

### 2.2. Video-Plus-Depth 3D Video

In this paper, we focus on the video-plus-depth representation of multiview 3D video which renders a 3D video using a 2D video stream and its associated depth map. This type is widely used because it provides a flexible representation of 3D and because it is compatible with existing coding and transmission technologies [3–8].

**Figure 2:**
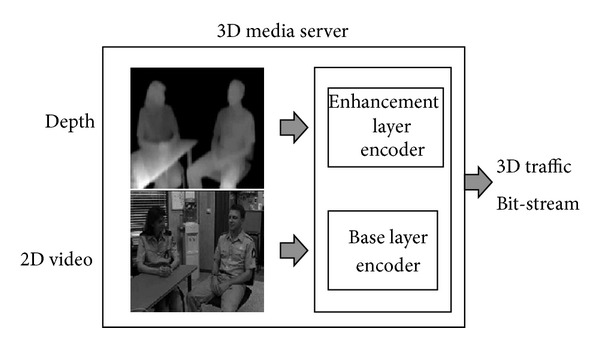
Layered coding approach of video-plus-depth 3D video.

In this format, the depth map includes 256 leveled grey images. It also contains depth information about the pixel positions of the associated 2D video. It can be acquired by a depth camera directly or can be extracted by a multiview image. The size of the depth map is related to the number of viewpoints of the 3D video. Because the resource requirements of 3D video applications based on the video-plus-depth scheme are high compared to those of 2D video applications, efficient compression techniques are required for 3D video. The layered coding approach can be effective with regard to this requirement. In the layered coding approach shown in Figure 2, the 2D video and the depth image sequence are encoded as the base layer and the enhancement layer, respectively. Existing 2D compression techniques are used to encode both the 2D video and the depth map sequence. After delivery, the base and enhancement layers are decoded as the 2D video and the depth image sequence on the receiver side. Before displaying 3D video on the display, the supplied 2D video and depth image sequences are converted into 3D video sequences using an image-warping technique known as DIBR.

## 3. Proposed Resource Allocation Strategy 

This section gives a detailed description of the proposed resource allocation strategy adaptive to 3D video over wireless systems. First, we describe the key idea of the proposed strategy to guarantee the QoS, formulating it as an optimization problem. Next, we present a suboptimal heuristic approach to solve this problem.

### 3.1. Description of the Proposed Strategy

There have been several definitions and measures pertaining to the QoS of a 3D A/V service [1–8]. Among them, we use continuity of service as the QoS measure of the 3D A/V service. Accordingly, a consecutive frame transmission in the MAC layer is required to guarantee the QoS. Moreover, an efficient resource allocation method is required due to the limited resources in wireless networks.

The key idea of the proposed resource allocation strategy is that the base layer traffic is given priority when assigning resources to guarantee the QoS. In this paper, we focus on 3D video traffic based on the video-plus-depth method, which consists of a 2D video stream and its associated depth map. As noted in Section 2.2, the 2D video and the depth map are separately encoded as the base layer and the enhancement layer, respectively. These two layers of data are transmitted together through a single connection. Given that the enhancement layer data contains valuable information with which to implement the 3D video, it should be considered as important. However, the base layer data is more important than the enhancement layer data with regard to the QoS, because the enhancement layer data plays a supporting role to convert the base layer data into 3D video. Assuming that eNB can transmit to a UE either the base layer data or the enhancement layer data on account of a shortage of resource, in such a case, if the UE receives only the enhancement layer data, it can no longer be provided with video streaming service. However, if the UE receives only the base layer data, it can avoid interruptions of its video streaming service, though 2D video is provided instead of 3D video. Therefore, we give priority to the base layer traffic, which can provide a 2D service by itself. This also helps to guarantee the QoS.

This idea is adopted in the scheduling procedure which works in the MAC layer of the eNB. As described in Section 2.1, the base layer data and the enhancement data are delivered from a 3D media server to the MAC layer buffer in the eNB. These two types of traffic are inserted into logically separated buffers. They are transmitted to the destination (the MAC layer of the UE) when resources are allocated by the scheduler at every slot time. The scheduling algorithm aims to achieve optimal resource allocation which maximizes the throughput of the 3D A/V traffic while guaranteeing the QoS. It is formulated as described below.

We assume that traffic whose destination is UE *i* is delivered to eNB at an average rate of packet/slot time. The packets are stored in a MAC buffer, *B*_*i*_. For the 3D A/V traffic, two buffers are assigned to the enhancement layer traffic and the base layer traffic, respectively. In this paper, we assume that the buffers are large enough to not to overflow. We stipulate that the amount of data in buffer *B*_*i*_ at the beginning of the* k*th slot is *x*_*i*,*k*_. From the result of scheduling, *u*_*i*,*k*_ is transmitted during* k*th slot. *u*_*i*,*k*_ depends on the allocated data rate at buffer *B*_*i*_. Then, the buffer is updated as

(1) $$\begin{array}{c} x_{i,k+1}=x_{i,k}+a_{i,k+1}-u_{i,k}, \end{array}$$

where *a*_*i*,*k*_ is the input traffic size in *B*_*i*_ during the* k*th slot. Let *D*_*i*_ be the average queuing delay for *B*_*i*_. *D*_*i*_ is related to the average buffer length via Little's theorem [15] and is described as

(2) $$\begin{array}{c} D_{i}=\frac{1}{\lambda }E\left[x_{i,k}\right], \end{array}$$

where *λ* = *E*[*a*_*i*,*k*_] is the average packet arrival rate. Because 0 ≤ *u*_*i*,*k*_ ≤ *x*_*i*,*k*_, the smallest average delay of the* k*th slot is achieved when *u*_*i*,*k*_ = *x*_*i*,*k*_ and the average queuing delay becomes *D*_*i*_ = 1.

Let *G*_*i*,*n*_ be the channel gain, *N*_*i*,*n*_ the total noise power spectral density, and *p*_*i*,*n*_ the allocated power for user *i* to subcarrier *n*. In this formulation, we consider an OFDMA based wireless system. We assume that M-QAM modulation is applied with a BER requirement. The signal-to-noise ratio (SNR) SNR_*i*,*n*_ is given by

(3) $$\begin{array}{c} \text{SN}{\text{R}}_{i,n}=\frac{{\left|G_{i,n}\right|}^{2}}{N_{i,n}\cdot \Gamma }, \end{array}$$

where Γ = −ln⁡⁡(5 · BER)/1.5 [16]. In addition, the capacity of user *i* on subcarrier *n* is normalized by

(4) $$\begin{array}{c} r_{i,n}=\ln\left(1+p_{i,n}\cdot \text{SN}{\text{R}}_{i,n}\right). \end{array}$$

The instantaneous data rate of user *i* can then be described as

(5) $$\begin{array}{c} R_{i}=\overset{N}{\underset{n=1}{\sum }}w_{i,n}\ln\left(1+p_{i,n}\text{SN}{\text{R}}_{i,n}\right), \end{array}$$

and the number of resource elements (RE's) required to support *R*_*i*_ while transmitting on subcarrier *n* is *s*_*i*_. *w*_*i*,*n*_ is the subcarrier allocation index; it has a value of 1 when subcarrier *n* is allocated to user *i*. Otherwise, it has a value of 0.

Because our goal is throughput maximization while guaranteeing the QoS, the scheduling problem can be written as shown below:

(6) Maximize  OP(w,p)=max⁡wi,n,pi,n ∑i=1Iln⁡Ri

(7) subject to   Dib−1≤Dth,  Dib≥1 ∀i

(8)  ∑i=1I ∑n=1Npi,n≤PT  pi,n≥0 ∀i,n

(9)  ∑i=1Iwi,n≤1  wi,n≥0 ∀i,n

(10)  ∑i=1Isi≤Smax⁡.

Here, *I* is the total number of UEs and *D*_*i*_^*b*^ is the average queuing delay of the buffer which contains the base layer traffic for the UE* i. D*_th_ is the threshold delay and *S*_max⁡_ is the total number of RE's within a slot. *w* = [*w*_*i*,*n*_]_*I* × *N*_, *p* = [*p*_*i*,*n*_]_*I* × *N*_, and *P*_*T*_ are the total available transmission power for eNB. Note that OP(*w*, *p*) is neither convex nor concave with respect to (*w*, *p*). Although *w*_*i*,*n*_ is defined to obtain a value of either 0 or 1, it is permitted to be a real number between 0 and 1 to make the problem tractable.

Equation (6) represents the total throughput computed by summing up the logarithmic user data rate assigned to each UE. There are four constraints. Equation (7) indicates that the queuing delay for the base layer traffic should not exceed the threshold delay bound, as the data in the buffer is discarded if it fails to be transmitted before the threshold delay bound. Consequently, this constraint implies that the base layer traffic is given priority in when assigning resources. This constraint is employed to guarantee the QoS.

Equation (8) indicates that the sum of the power allocated to each UE should not exceed the total available power. Equation (9) indicates that only one subcarrier can be allocated to a UE. Equation (10) indicates that the sum of the allocated RE of each UE should not exceed the total number of RE's. These three constraints are used for throughput maximization.

To maximize OP(*w*, *p*), subcarrier *n* should be allocated to user i*. This is expressed as follows:

(11) $$\begin{array}{c} i^{*}=\arg\underset{i}{\max}\frac{r_{i,n}}{R_{i}}. \end{array}$$

In addition, the allocated power of user i* with subcarrier *n* is given as

(12) $$\begin{array}{c} p_{i^{*},n}=\max\left\{f_{i}^{*}-\frac{1}{\text{SN}{\text{R}}_{i^{*},n}},0\right\}, \end{array}$$

where *f*_i_* is the water-filling level of user i* [17].

### 3.2. Heuristic Approach

Given that the scheduling problem formulated in Section 3.1 is a NP-hard problem, we adopt a suboptimal heuristic approach to solve this problem practically. The details of the heuristic algorithm are shown in Algorithm 1.

In this heuristic algorithm, *x*_*i*_^*b*^ indicates the amount of base layer traffic within the buffer and *x*_*i*_^*e*^ indicates the amount of enhancement layer traffic within the buffer. *T*_*i*_ is the requested traffic size for user *i*, and *d*_*i*_^*b*^ and *d*_*i*_^*e*^ indicate the queuing delay of base layer packets and enhancement layer packets, respectively. *S*_*a*_ is the total number of assigned RE's at one time instance and Δ is the time margin for priority adjustments.

A base layer packet and an enhancement layer packet for a 3D video traffic are delivered from a 3D media server to logically separated MAC layer buffers in eNB. When a packet is generated and stored in the buffer, the index value of the buffer increases by one. They are transmitted to the MAC layer of the destination UE when resources are allocated by the scheduler at every slot time. The heuristic algorithm in Algorithm 1 is involved in the scheduling process.

In the heuristic algorithm, a user whose average SNR is higher has priority in resource allocation. So, the user who has the highest average SNR gets resource first. The scheduler finds and allocates the best subcarrier which maximizes *r*_*i*,*n*_. According to the user's requested traffic size and allocated subcarrier's capacity, RE's are allocated. Then, the heuristic algorithm decides how to share the allocated RE's between buffered traffic.

The basic rule is that the base layer traffic is processed first after which the enhancement layer traffic is handled only when there are remaining resources. However, we can adjust the level of priority for the base layer traffic by adjusting the value of Δ. If the queuing delay of the base layer packet does not exceed the adjusted threshold delay bound and the queuing delay of the enhancement layer packet exceeds the adjusted threshold delay bound, resources are used for the transmission of the enhancement layer packet. If we use a higher value of Δ, higher priority to the base layer traffic is given. Δ has a value between 1 and *D*_th_.

After allocating resources for the subcarrier and the RE, the power is also assigned. Assuming that all subcarriers have equal power, *p*_*i*,*n*_ = *P*_*T*_/*N* for any *i* and *n*. In this case, the total transmission power is given by

(13) $$\begin{array}{c} P_{i}=\frac{\left|S_{i}\right|P_{T}}{N}, \end{array}$$

where *S*_*i*_ is a set of subcarrier allocated to user *i* and |*S*_*i*_| is the cardinality of *S*_*i*_. From (12), power allocation to user *i* on the subcarriers in *S*_*i*_ can be calculated by means of a water-filling technique. Then, if we redescribe *f*_*i*_ as

(14) fi=pi,n+1SNRi,n=1|Si|(Pi+∑n∈Si1SNRi,n), ∀n∈Si

and substitute this into (12), we can obtain *P*_*i*,*n*_ [17].

## 4. Performance Evaluation 

To evaluate the performance of the proposed method, a simulation is carried out. In this section, we describe the simulation environment first and then evaluate the simulation results using three performance measures.

### 4.1. Simulation Environment

To evaluate the proposed strategy, we perform a series of simulations. In the simulations, we consider a single cell model which consists of one eNB and a number of UEs. We assume that the cell radius is 1 km and the carrier frequency of eNB is 1.9 GHz. We also assume that the total available transmit power of eNB is 10 W and the noise power is −100 dB. The path loss model given below is used for the channel between eNB and the UE [18]:

(15) $$\begin{array}{c} L=128.1+37.1\log d\left(\text{dB}\right). \end{array}$$

Shadowing is lognormally distributed with a mean of 0 dB and a standard deviation of 8 dB. The target BER is assumed to be 10^−4^.

Because we consider an OFDMA based wireless system, we also set several OFDMA parameters. The system bandwidth is assumed to be 10 MHz and the slot time is set to 0.5 ms. We adopt the channel structure of the 3GPP Ericsson model [19, 20]. To mitigate the long simulation time, we assume that the maximum number of RE's during a slot *S*_max⁡_ is 12 and the number of subcarriers is 6. The modulation method and coding rate are changed according to the SNR. The modulation and coding scheme (MCS) has nine levels, as shown in Table 1.

UEs are uniformly distributed in a cell and each UE is served only one type of traffic. In the simulation, we use two traffic models: 3D A/V traffic models and web traffic models. We assume that 10% of the UEs are served WEB services and 90% of UEs are served the 3D A/V service.

**Figure 3:**
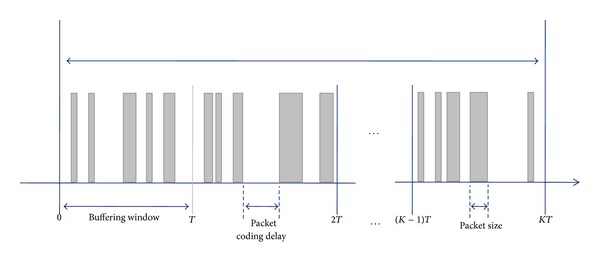
3D A/V traffic model.

The traffic model of 3D A/V used in this simulation is formulated based on the streaming video traffic model which is used for IEEE 802 systems [21]. Because the base layer traffic of 3D A/V is identical to the characteristics of the streaming 2D video traffic, we use the streaming video traffic model as the basis of the 3D traffic model. We then append the enhancement layer traffic model which is changed according to the number of 3D viewpoints. In this 3D A/V traffic model, each frame consists of a constant number of packets. The packet size and its arrival time within one frame are defined as a truncated Pareto distribution.  Figure 3 shows the 3D A/V traffic model and Table 2 shows its parameter values.

In Figure 3, *T* represents the interarrival time between the beginnings of each frame and the packet coding delay is determined by the interarrival time between packets in a frame, as shown in Table 2. The parameter *β* of the packet size of the enhancement layer changes according to the number of 3D viewpoints. For 8-viewpoint 3D video traffic, *β* is 0.45 [22]. For 16-viewpoint and 32-viewpoint 3D video traffic, *β* is 0.9 and 1.8, respectively.

WEB traffic has a form similar to the ON/OFF model. WEB traffic consists of the main object comprising the web page and several embedded objects. They are transmitted if a web page is requested.  Table 3 shows the WEB traffic model.

We also assume that the buffer of UE has infinite capacity. The maximum delay bound of 3D A/V frame *D*_th_ is set to 250 ms and the time margin for priority adjustment Δ is also set to 250 ms.

### 4.2. Numerical Results

**Figure 4:**
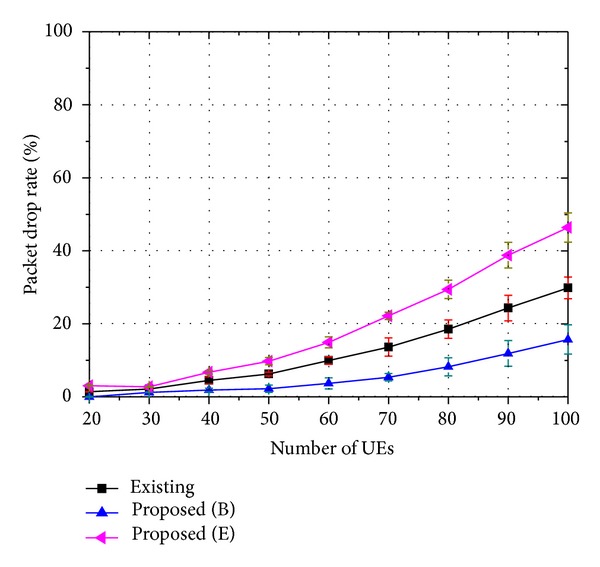
Packet drop rate of 8-viewpoint 3D A/V traffic (*β* = 0.45).

**Figure 5:**
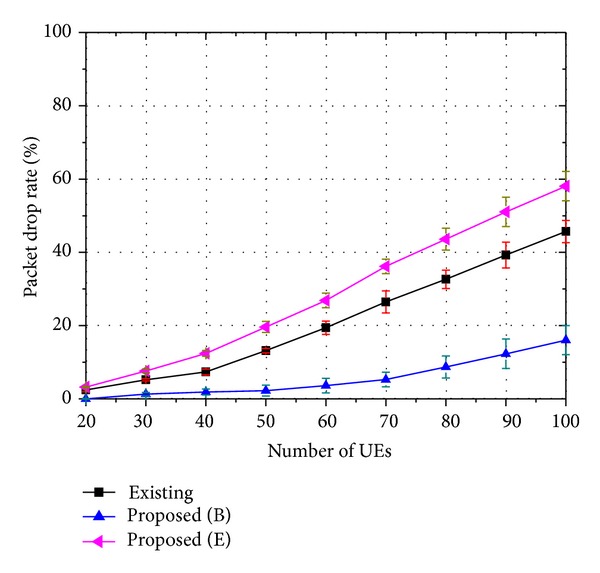
Packet drop rate of 16-viewpoint 3D A/V traffic (*β* = 0.9).

**Figure 6:**
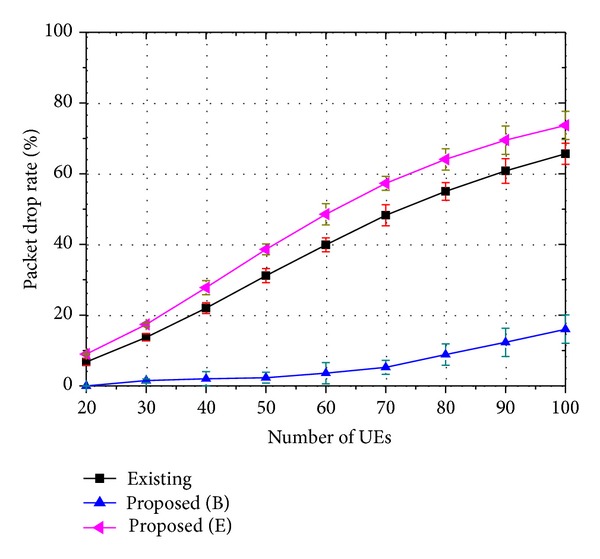
Packet drop rate of 32-viewpoint 3D A/V traffic (*β* = 1.8).

The performances of the proposed strategy are evaluated through the packet drop rate, the service success rate, and the QoS level. Figures 4, 5, and 6 show the packet drop rate when 8-viewpoint 3D video, 16-viewpoint 3D video, and 32-viewpoint 3D video are served, respectively. A packet in a buffer drops when it is unable to be transmitted within the maximum delay bound. In these figures, the proposed strategy is compared with an existing strategy. A similar scheduling algorithm is used in both the existing strategy and the proposed strategy, but they handle 3D traffic in different ways. The proposed strategy places higher priority on the base layer traffic. On the other hand, the existing strategy treats the base and the enhancement layer traffic in the same manner. In the figures, the label “Existing” indicates the packet drop rate of 3D traffic when the existing strategy is employed. The label “Proposed (B)” indicates the packet drop rate of the base layer traffic when the proposed strategy is employed. The label “Proposed (E)” indicates the packet drop rate of the enhancement layer traffic when the proposed strategy is employed.

As shown in Figures 4–6, the base layer traffic has a lower packet drop rate compared to the enhancement layer traffic. In addition, the packet drop rate of the enhancement layer traffic increases as the ratio of the enhancement layer traffic becomes higher. On the other hand, the packet drop rate of the base layer traffic shows little change, because the proposed strategy prioritizes the base layer traffic. In fact, the packet drop rate of the existing strategy is similar to the sum of the packet drop rates of the base layer traffic and the enhancement layer traffic, as the same level of resources is allocated regardless of whether it uses the proposed strategy or the existing strategy.

**Figure 7:**
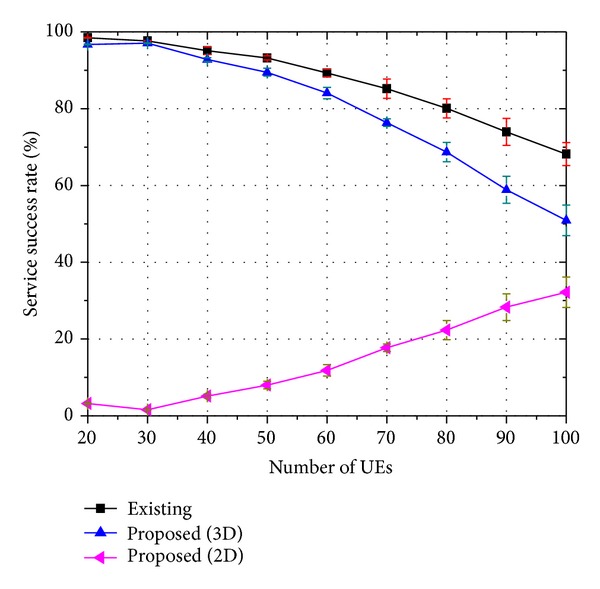
Service success rate of 8-viewpoint 3D A/V traffic (*β* = 0.45).

**Figure 8:**
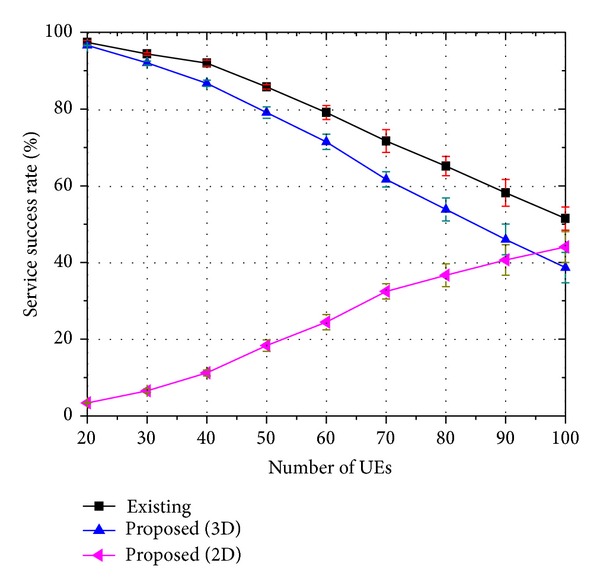
Service success rate of 16-viewpoint 3D A/V traffic (*β* = 0.9).

**Figure 9:**
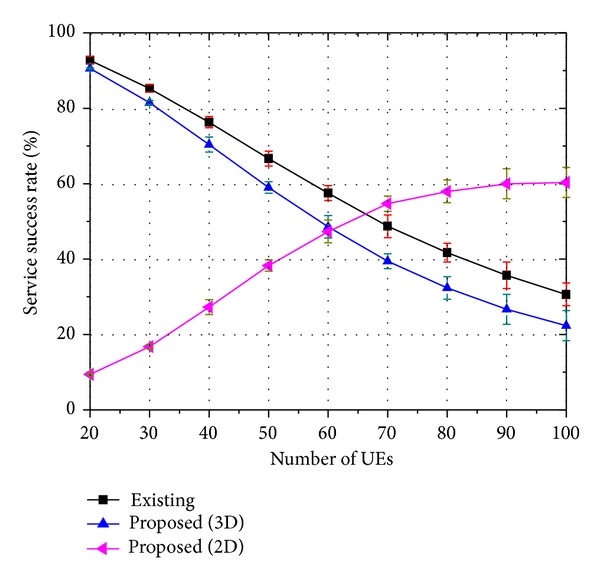
Service success rate of 32-viewpoint 3D A/V traffic (*β* = 1.8).

Figures 7, 8, and 9 show the service success rate of the proposed strategy. We defined the service success rate as the rate received by an end user successfully for a type of service. Consequently, the service success rate is closely related to the QoS. In the figures, the label “Existing” indicates the rate at which 3D service is successfully served when the existing strategy is employed. The label “Proposed (3D)” indicates the rate at which 3D service is successfully served when the proposed strategy is employed, indicating that both the base layer traffic and the enhancement layer traffic are successfully transmitted to the UE and the end user can watch 3D video. The label “Proposed (2D)” indicates the rate at which 2D service is successfully served when the proposed strategy is employed. Thus, only the base layer traffic is successfully transmitted to the UE and the end user can only watch 2D video.

Therefore, it is considered as “Proposed (3D)” if both the base layer traffic and the enhancement layer traffic are well transmitted. However, it is considered as “Proposed (2D)” if the enhancement layer traffic cannot be delivered due to a lack of resources and if only the base layer traffic is transmitted well. It is regarded as a service failure if both the base layer traffic and the enhancement layer traffic cannot be delivered or if only the enhancement layer traffic is delivered well.

When there are a small number of users, nearly all users can receive both the base layer traffic and the enhancement layer traffic successfully. As the number of users increases, the number of users who can receive only the base layer traffic increases because the proposed strategy places priority on the base layer traffic. Therefore, the service success rate of “Proposed (2D)” increases and the service success rate of “Proposed (3D)” decreases with an increase in the number of users.

**Figure 10:**
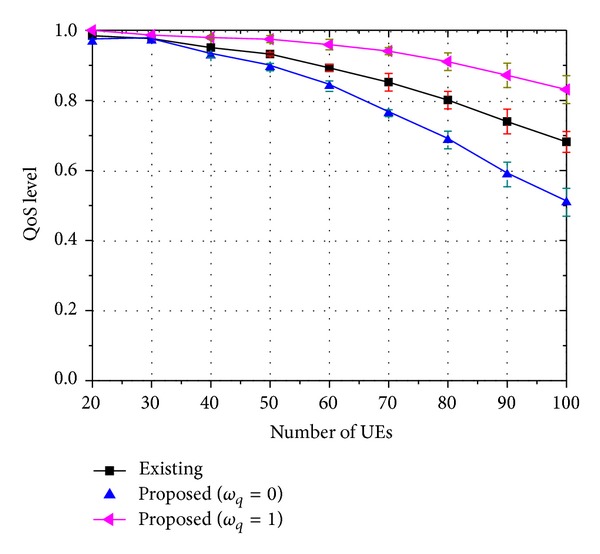
QoS level of 8-viewpoint 3D A/V traffic (*β* = 0.45).

**Figure 11:**
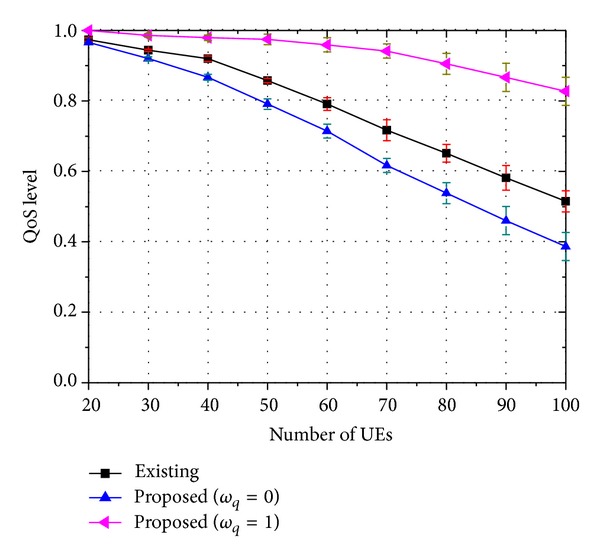
QoS level of 16-viewpoint 3D A/V traffic (*β* = 0.9).

**Figure 12:**
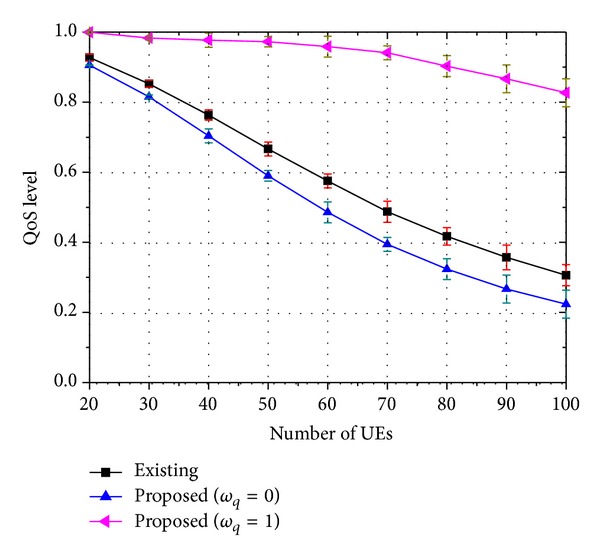
QoS level of 32-viewpoint 3D A/V traffic (*β* = 1.8).

The figures show that the 3D service success rate of the proposed strategy is similar to that of the existing strategy. However, if we assume that 2D video watching is also regarded as successful service, the proposed strategy outperforms the existing strategy. The meaning of these results can be clarified when we consider them as follows: let *ψ*_3D_ be the success rate of the 3D service and *ψ*_2D_ the success rate of the 2D service. The QoS level *ϖ* is then defined as

(16) $$\begin{array}{c} \varpi =\frac{\psi_{3\text{D}}+\omega_{q}\cdot \psi_{2\text{D}}}{100}, \end{array}$$

where *ω*_*q*_ is the user's satisfaction weight for the 2D service compared to that for the 3D service. *ω*_*q*_ has a value between 0 and 1. *ω*_*q*_ = 1 indicates that a 3D user is fully satisfied even when watching 2D video instead of 3D video. Additionally, *ω*_*q*_ is zero when a 3D user is fully disappointed if he watches 2D video instead of 3D video. The QoS level also has a value between 0 and 1. A value of 1 indicates a state of satisfaction and a value of 0 indicates a state of dissatisfaction. In Figures 10, 11, and 12, the pause-less and flawless execution of not only 3D video but also 2D video are regarded as a successful service. From the figures, the QoS level of the proposed strategy outperforms that of the existing strategy. As these strategies allocate similar amounts of resources to 3D traffic, we can conclude that the proposed strategy utilizes the resources more efficiently—in terms of the QoS. This result stems the use of the base layer traffic which contains 2D video information. Successful transmission of this information can improve the QoS level. Of course, if the enhancement layer traffic and the base layer traffic are successfully transmitted together, the user will be completely satisfied. However, complete transmission of the base layer information alone can also improve the QoS level.

From these numerical results, it is clear that the proposed strategy can guarantee better QoS compared to the existing strategy. The advantage of the proposed strategy becomes more evident as the rate of the enhancement layer traffic increases, or when the number of viewpoints of the 3D traffic grows.

## 5. Conclusions

In this paper, we propose a novel resource allocation strategy for 3D video over a wireless system to guarantee the QoS. The proposed strategy focuses on the relationship between 2D video and the depth map and handles them with different priorities. Performance evaluations show that our strategy is a good choice to guarantee better QoS. In terms of several performance measures such as the packet drop rate, the service success rate, and the QoS level, our strategy is shown to outperform an existing strategy. Moreover, the advantage of the proposed strategy increases as the number of viewpoints of 3D video is increased. Therefore, we expect that the proposed strategy can be very useful for more realistic 3D traffic delivery.

## Figures and Tables

**Algorithm 1:**
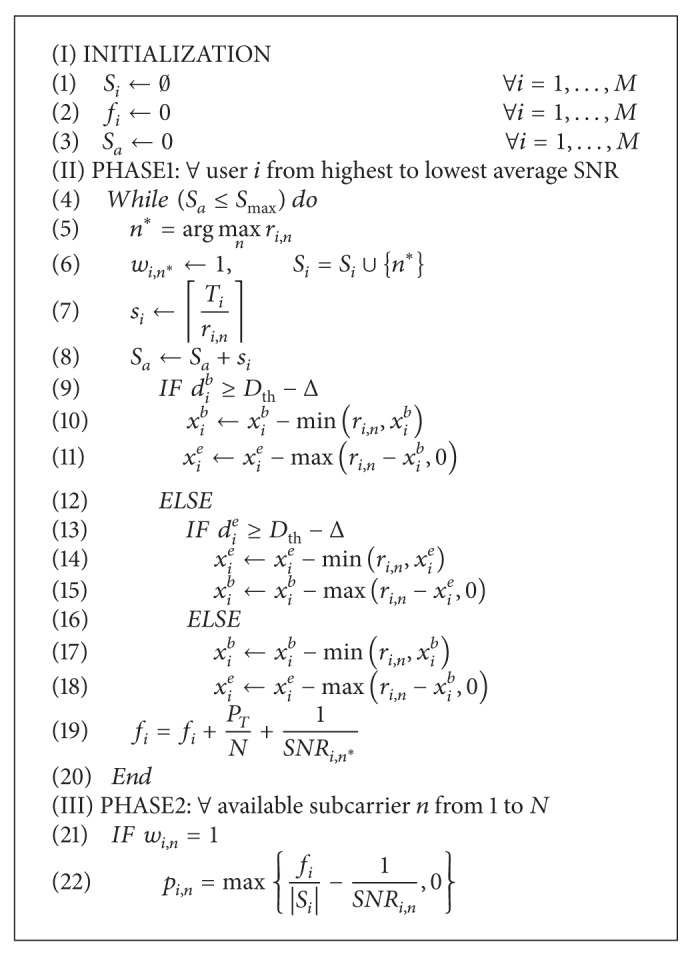
Heuristic algorithm.

**Table 1:** MCS level and data rate.

MCS level	Min ≤ SNR ≤ Max	Modulation scheme	Coding rate	Data rate (bit/slot)
M_1_	SNR < 2 db	BPSK	1/4	43.75
M_2_	2 db ≤ SNR < 5 db	BPSK	1/2	87.5
M_3_	2 db ≤ SNR < 5 db	BPSK	3/4	131.25
M_4_	2 db ≤ SNR < 5 db	QPSK	1/2	175
M_5_	2 db ≤ SNR < 5 db	QPSK	5/8	218.75
M_6_	2 db ≤ SNR < 5 db	QPSK	3/4	262.5
M_7_	2 db ≤ SNR < 5 db	QPSK	7/8	306.25
M_8_	2 db ≤ SNR < 5 db	16QAM	1/2	350
M_9_	2 db ≤ SNR < 5 db	16QAM	9/16	393.75

**Table 2:** Parameters of the 3D A/V traffic model.

Information	Parameter
Interarrival time between the beginnings of each frame (ms)	(Deterministic) 100
Number of packets in a frame	(Deterministic) 8
Packet size of the base layer (byte)	Truncated Pareto (mean = 50, max = 125), *K* = 20, *α* = 1.2
Packet size of the enhancement layer (byte)	(Packet size of the base layer)∗*β*, *β* = 0.45, 0.9, 1.8
Interarrival time between packets in a frame (ms)	Truncated Pareto (mean = 6, max = 12.5), *K* = 2.5, *α* = 1.2

**Table 3:** WEB traffic parameters.

Information	Parameter
Main object size (byte)	Truncated Lognormal (mean = 10710, min = 100, max = 2,000,000)
Embedded object size (byte)	Truncated Lognormal (mean = 7758, min = 50, max = 2,000,000)
Number of pages	Truncated Pareto (mean = 5.64, max = 53), *K* = 2, *α* = 1.1, *m* = 55
Reading time (s)	Exponential (mean = 30)
Parsing time (s)	Exponential (mean = 0.13)
